# A research and development fund for new treatments for bacterial infections

**DOI:** 10.2471/BLT.20.282004

**Published:** 2020-12-01

**Authors:** Peter Beyer, Thomas B Cueni, Jeremy Knox, Felicitas Riedl

**Affiliations:** a Department of Global Coordination and Partnership, AMR Division, World Health Organization, Avenue Appia 20, 1211 Geneva 27, Switzerland.; b International Federation of Pharmaceutical Manufacturers and Associations, Geneva, Switzerland.; c Wellcome Trust, London, England.; d European Investment Bank, Luxembourg.

The current focus on the coronavirus disease 2019 (COVID-19) pandemic means a lack of attention to other health challenges, such as antimicrobial resistance. The setting up of the AMR Action Fund, which will invest in antibiotic research and development, is therefore an important development.

Unlike COVID-19, which caught the global community ill-prepared, the public health threat of antimicrobial resistance is well-known and has existed from the first instance of antibiotic use. Antimicrobial resistance also has considerable potential to cause death and economic loss. The World Bank reports that by 2050, antimicrobial resistance could cause low-income countries to collectively lose more than 5% of their gross domestic product and push 28 million more people into poverty by 2050.^1^

For several years, the global health community and politicians have been warning of the dangers posed by antimicrobial resistance, and particularly the lack of new antibiotics to replace those that are losing their efficacy due to increasing bacterial resistance.^2^ Most of the major pharmaceutical companies have abandoned antibiotic research and development, and private investment has largely vanished after the bankruptcy of several biotech companies.^3^ For companies working on promising new drug candidates, their efforts may falter after they progress into costly Phase II and III trials.

The public sector is trying to compensate for the loss of investment in research and development of new antibiotics by setting up the Global Antibiotic Research and Development Partnership (GARDP) and the Combating Antibiotic-Resistant Bacteria Biopharmaceutical Accelerator (CARB-X), among others. While such initiatives have strengthened the preclinical and clinical pipeline,^4^ overall public investment cannot compensate for the current loss of private investment.

The AMR Action Fund was launched in July 2020,^5^ with nearly 1 billion United States dollars provided by over 20 pharmaceutical companies, with the aim of bringing two to four novel antibiotics to market by the end of the decade. The funding and know-how provided by the participating pharmaceutical companies will help address the critical challenge of clinical development. Although initially developed as an industry initiative, the fund’s architecture was created with the European Investment Bank (EIB), Wellcome Trust and the World Health Organization (WHO). Through this collaboration, the fund benefited from groundwork that done by WHO and the EIB to create an impact investment fund and an underlying financial model to demonstrate its feasibility.^6^ The fund anticipates that the EIB (supported by the European Commission) and philanthropic organizations will join as investors.

The AMR Action Fund will invest in biotech companies developing innovative antibacterial treatments (in Phase II and beyond) that target public health priorities. The fund will focus on new treatments that are suitable for all countries and can reach a maximum of patients while ensuring that new antibiotics are used appropriately to conserve their effectiveness for as long as possible.^7^

However, the fund cannot solve the underlying problem: the current market environment makes it very difficult for new antibiotics to survive economically. We need to rethink how new antibiotics are valued and purchased. The idea of globally coordinated pull incentives has been widely advocated and discussed in the Group of Seven and the Group of Twenty, but only a few countries have so far responded to these calls for action.

The United Kingdom of Great Britain and Northern Ireland has initiated a pilot project under which the National Health Service in England is procuring two antibiotics not by paying for volume, but by paying for their availability, thus delinking price from volume. In the United States of America, the Pioneering Antimicrobial Subscriptions to End Upsurging Resistance (PASTEUR) Act,^8^ if approved, will provide the market authorization holder of new antibiotics that fulfil certain conditions with a minimum guaranteed income, in addition to providing grants for appropriate use and better surveillance at hospitals. Germany has revised the way it assesses reserve antibiotics, to better recognize the value of novel antibiotics. All these initiatives are going in the right direction and will contribute to making the antibiotic market more sustainable, but more are needed. 

The AMR Action Fund is only a bridging solution: it will buy the world time to address the root cause of the lack of investment in antibiotic research and development. If governments do not address the market environment, new antibiotics will fail to reach patients who need them. More countries need to examine how they procure and use antibiotics with the aim of making these products globally available and keeping them in the market. 
